# Polymer ionic liquid network: a highly effective reusable catalyst for one-pot synthesis of heterocyclic compounds†

**DOI:** 10.1039/c8ra08712a

**Published:** 2018-12-19

**Authors:** Xinjuan Li, Shangyue Wang, Kai Wang, Xianbin Jia, Zhiguo Hu

**Affiliations:** Henan Key Laboratory of Green Chemistry, Collaborative Innovation Center of Henan Province for Green Manufacturing of Fine Chemicals, Key Laboratory of Green Chemical Media and Reactions, Ministry of Education, School of Chemistry and Chemical Engineering, Henan Normal University Xinxiang 453007 P. R. China xinjuanli2009@163.com zghu@htu.cn

## Abstract

Significant efforts have been devoted to developing immobilized chiral catalysts with high activity, selectivity, and stability. In this present study, a new heterogeneous proline catalyst system was prepared based on strong noncovalent interactions between polymer ionic liquid (PIL) and l-proline. First, pyridine PILs, which can complex with l-proline monomers through noncovalent interactions, were synthesized using reversible addition–fragmentation chain transfer (RAFT) polymerization. The polymer network-supported chiral catalysts were obtained following further free radical polymerization. Different structures were formed in response to different ratios of PIL and chiral monomer, as well as different PIL anions, in the reactions. The new formed layer structures and synergic effects of PIL resulted in heterogeneous catalysts with high catalytic activity and enantioselectivity, thus endowing them with better catalytic performance for the one-pot synthesis of heterocyclic compounds compared to homogeneous catalytic systems. These catalytic systems were able to be reused and recycled five times with no discernible loss in catalytic activity and enantioselectivity. l-Proline was efficiently loaded onto the polymer network simply based on supramolecular interactions, providing a novel method of synthesizing high performance supported catalysts for organic reactions.

## Abstract 2

Significant efforts have been devoted to developing immobilized chiral catalysts with high activity, selectivity, and stability.

## Graphical abstract

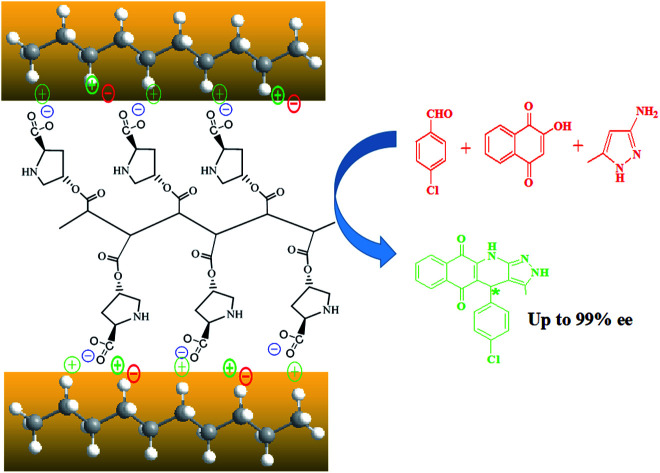

## Introduction

Asymmetric aldol reactions are important and well-known C–C bond-forming reactions in organic synthesis and are widely used in the agrochemical, pharmaceutical, and fine chemical industries.^1^ Multicomponent reactions (MCRs), which combine three or more reagents in a one-pot process, require only a single step and simple experimental conditions to generate a final product. MCRs used in combination with asymmetric aldol reactions are powerful tools with which to create a series of heterocycles that have already attracted extensive attention in organic synthesis and drug discovery.^2^

Since List *et al.* first reported proline can efficiently catalyze direct asymmetric aldol reactions, proline and its derivatives have been extensively developed and applied in asymmetric catalytic reactions.^3–9^ However, these methods have some disadvantages, such as limited substrate scope and difficult separation and recycling, that prevent them from further development and application in industry.^10^ Immobilization of l-proline and its derivatives effectively overcomes these disadvantages and has been the focus of considerable work in recent years.^10–16,31–34^ Supporting solids commonly used include polymer,^17,18^ silica,^19,21^ ionic liquid,^23–29^ merrifield resin,^22^ and magnetite.^20^ It remains a challenge to prepare highly efficient and recyclable chiral catalyst. Ionic liquids have been used as carriers in catalyst loading,^24,30^ where they can act as regulators to improve the efficiency and reusability of chiral catalyst in direct asymmetric aldol reactions.^25,26^ However, the separation of IL-tagged catalyst is an undesirable process. Polymer ionic liquid (PIL) is a polymer with an ionic liquid repeat unit that possesses the unique characteristics of polymers and ionic liquids containing anions and cations.^2^ Compared with small molecule ionic liquids, it is easier to use PIL to prepare effective load catalytic systems due to its variable long-chain structure. Our team has prepared nanoparticle-supported l-prolinamide catalyst based on PIL using *in situ* ionic complexation.^2^ The PIL-modified solid catalytic systems have advantages in terms of separating and accelerating asymmetric reactions.

**Fig. 1:**
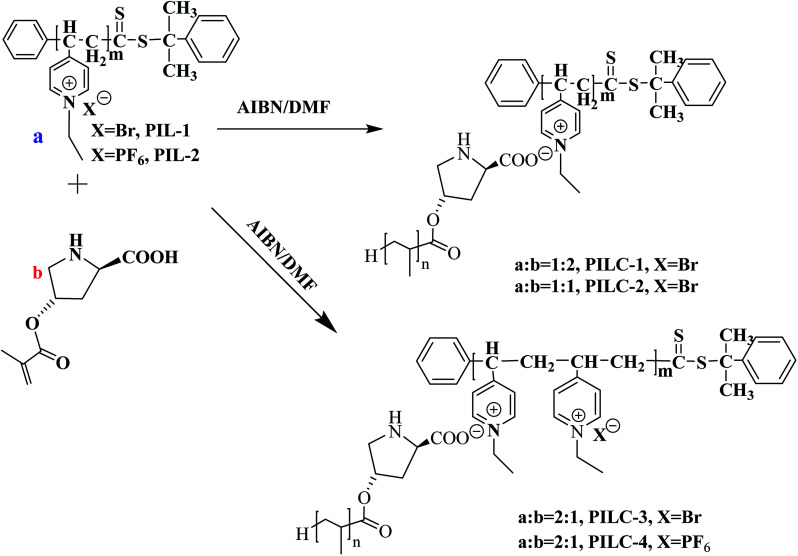
Synthesis of heterogeneous catalyst-supported l-proline based on supramolecular interactions and free radical polymerization.

In this present study, we illustrate a novel concept for the preparation of the heterogeneous proline catalyst system which was prepared based on the strong noncovalent interactions between PIL and l-proline. Pyridine PILs with well-controlled architectures formed *via* reversible addition–fragmentation chain transfer (RAFT) polymerization can create strong noncovalent bonds with l-proline monomer due to the non-covalent interactions between l-proline and PIL.^35^ Furthermore, the catalytic networks were formed through one step of free radical polymerization (Fig. 1). The obtained catalysts were characterized using Fourier transform infrared spectra (FT-IR), scanning electron microscopy (SEM), X-ray diffraction (XRD), X-ray photoelectron spectroscopy (XPS), and elemental analysis. The influences of different proportions of PIL and proline monomer, as well as PIL anion structure, on catalytic activity and asymmetric selectivity were investigated through aldol reactions and MCRs. It was found the PIL complex provides a new layer structures and synergic effects that increase the catalytic performance of the heterogeneous l-proline catalyst. In this present study, l-proline was efficiently loaded onto the polymer network through noncovalent interactions and free radical polymerization, thus providing a new approach to synthesizing high performance supported catalysts for use in organic reactions.

## Experimental section

### Materials and methods

The chemicals 2-chlorobenzaldehyde, 4-nitrobenzaldehyde, and 4-acetamido benzaldehyde were purchased from TCI. Azobisisobutyronitrile (AIBN) was recrystallized in ethanol. *N*-Dimethylformamide (DMF) was purified by distillation after drying. Aromatic aldehyde and 4-vinyl pyridine (4-VP) were purified by distillation. l-Proline monomer and cumyl dithiobenzoate (CDB) were prepared according to a previous publication.^2^

^1^H NMR spectra were obtained using an NMR spectrometer (Bruker 400 MHz). A Thermo FLASH 1112 elemental analyzer was used for elemental analysis. Infrared spectroscopy and HPLC were performed on a Fourier transform-infrared spectrometer (Nicolet NEXUS) and an Agilent TM 1100, respectively. The molecular weight distribution (PDI = *M*_w_/*M*_n_) and molecular weight of the synthesized polymer were measured by gel permeation chromatography using a Waters 1515 apparatus, DMF as the eluent at a flow rate of 1.0 mL min^−1^, and polystyrene samples as standards. Field emission scanning electron microscopy (NovaNano SEM450) were used to characterize the morphology of the catalyst. XPS spectra were obtained on a VG ESCALAB MK II spectrograph.

### Preparation of heterogeneous catalyst-supported l-proline

#### Preparation of PILs

CDB (20.7 mg, 0.076 mmol), 4-VP (0.40 g, 3.85 mmol), AIBN (3.1 mg, 0.019 mmol), and DMF (5 mL) were combined in a 10 mL round-bottom flask. After stirring for 10 min, a clear solution was obtained. Oxygen was completely removed from the system with nitrogen and then the flask was sealed and incubated at 65 °C with stirring. After allowing the reaction to proceed for 48 h, the product was diluted in ether, centrifuged, washed with ether to remove 4-VP monomer, and dried at 40 °C to obtain P4VP with the yield of 66%.

After dissolving P4VP (0.1 g, 0.95 mmol) in 3 mL chloroform, 9.5 mmol bromoethane was added to the flask and the mixture was heated to 60 °C for 48 h. After the mixture had cooled, the solid was thoroughly washed with chloroform to remove unreacted P4VP and the obtained solid was dried under pressure to create PIL-1. The average molecular weight was 5301 and the PDI = 1.29 (gel permeation chromatography analysis).

PIL-2 with hydrophobic anion (PF_6_^−^) was also prepared using a standard procedure. PIL-1 (29.30 g, 0.067 mmol) and KPF_6_ (12.33 g, 0.067 mmol) were dissolved in 100 mL distilled water, which formed a clear solution, and then stirred at 25 °C for 5 h until an insoluble oily substance was formed, which was collected by centrifugation. The crude product was washed first with water and then ether and dried at 60 °C under a vacuum.

### Synthesis of poly(ionic liquid) complex networks

Poly(ionic liquid) complexes (PILCs) were prepared as follows: 0.02 g PIL-1 (2 mmol) and 0.40 g l-proline monomer (2 mmol) were dissolved in 10 mL of DMF and then 2.2 mg AIBN (0.0134 mmol) were added. In order to remove oxygen from the system, degassing was carried out using five freeze and thaw cycles and then the sealed bottle was put into an oil bath at 75 °C for 48 h. The product was collected by centrifugation and washed with DMF and ethanol 5 times to remove unreacted monomer and the free polymer. The resulting light blue product was dried at 40 °C for 48 h to obtain PILC-1 with the yield of 80%.

PILC-2, which contained a molar ratio of PIL-1 to proline monomer of 2 : 1, and PILC-3, which contained a molar ratio of PIL-1 and proline monomer of 1 : 2, were synthesized in a manner similar to PILC-1, except using different molar ratios of PIL and proline monomer.

Synthesis of PILC-4 was similar to PILC-3 except PIL-2 (PF_6_^−^) was used.

### PILC application in asymmetric reactions

PILCs were applied in asymmetric aldol reactions of ketone with 2-nitrobenzaldehyde.

Acting as catalysts, PILCs (10 mol%, 0.01 mmol proline content) were added to a mixture of acetone (104 μL, 1.0 mmol) and 4-nitrobenzaldehyde (0.25 mmol, 38 mg) in 1 mL of the corresponding solvent. The mixtures were stirred at different temperatures and monitored by thin-layer chromatography (TLC) until the reactions were complete. The reaction mixtures were isolated by centrifugation and the PILCs were washed with methanol. The solids were dried under a vacuum for the next cycle. The aqueous layers were extracted with EtOAc and then dehydrated with MgSO_4_. After evaporation of the solvent, the crude products were separated and purified by column chromatogram (petroleum ether/EtOAc = 4 : 1, v/v) to yield the desired products.

### PILCs applied in MCRs

Acting as catalysts, PILCs (20 mol%, 0.2 mmol proline content) were added to a mixture of 2-hydroxy-1,4-naphthoquinone (1 mmol) and aldehyde (1 mmol) in the corresponding 1 mL solvent. The mixture was heated to 80 °C with stirring for 30 minutes. After the addition of 1 mmol 3-amino-5-methylpyrazole, the reaction continued with stirring until complete as monitored by TLC. The resulting solid was filtered and purified using ethanol. The PILC catalysts were then dissolved in tetrahydrofuran, filtered, and washed with ethanol for the next cycle. The aqueous layer was then evaporated to yield the desired product.

## Results and discussion

### Preparation of the heterogeneous catalyst-supported l-proline

In our previous research, PILs were successfully used for highly sensitive detection of amine acid in solution. The biosensitivity of PIL was dependent on the carboxyl and secondary amine groups of l-proline, which can form ionic bonds and hydrogen bonds with PIL.^34^ The polymer network supported l-proline was obtained by mixing PIL with l-proline monomer in DMF solution and further free radical polymerization. In addition, we also reported that PIL had synergic effects and improved the performance of the catalytic system.^3^ Therefore, we synthesized a series of complex networks by modifying the reaction ratios of PIL and l-proline monomer and PIL anion to study the influence of ionic liquid polymer on the performance of the catalysts.

### Sample characterization

**Fig. 2:**
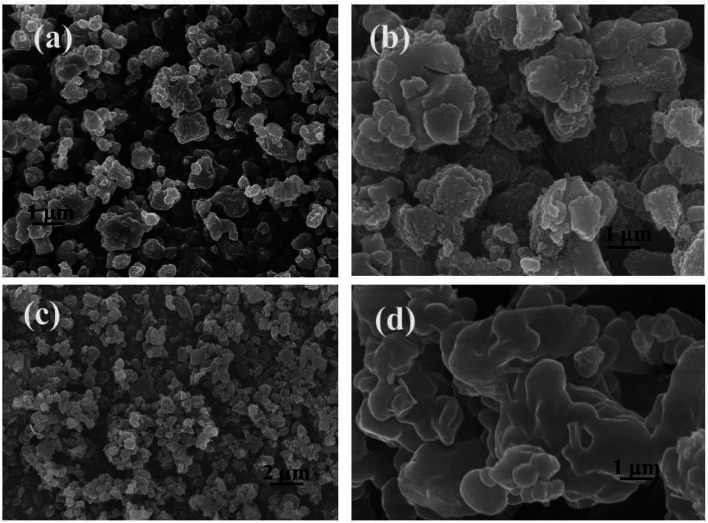
SEM images of catalyst (a) PILC-1, (b) PILC-2, (c) PILC-3, and (d) PILC-4.

Fig. 2 presents the microstructure and morphological evolution of the heterogeneous catalyst. SEM images of PILC-1, which appeared as sheets with a polygonal morphology, a length of approximately 800–900 nm, are shown in Fig. 2a. Fig. 2b presents the microscopic structure and morphological evolution of PILC-2, where also formed a heterogeneous layer structure. When more PIL added than proline monomer was added (as in Fig. 2c, where the molar ratio was 1.25 for PIL and proline monomer, as shown in Table 1) as for PILC-3, the PILC matrix formed a heterogeneous layer structure. Overall, the individual proline polymers were homogeneously dispersed throughout the PIL network and the proline polymer was physically absorbed onto the surface of the PIL, assembling into a new structure during the simple process of radical polymerization. PILC-4 with PF_6_^−^ anion formed a homogeneous amorphous structure (Fig. 2d), where the proline polymer had fully permeated into the PIL matrix and formed no regular structure.

**Table 1:** Chemical composition of different PIL networks determined by elemental analysis

Sample	Yield (%)	N content (wt%)	S content (wt%)	l-Proline content^a^ (mmol g^−1^)	Chiral polymer mol ratio : PIL^b^
PIL-1		5.24	0.71	0	
PILC-1	87	6.23	0.47	1.99	1.25 : 1
PILC-2	86	5.89	0.37	2.23	1 : 1.16
PILC-3	80	6.09	0.33	2.61	1 : 1.51
PILC-4	92	5.65	0.43	1.70	1 : 1.26
PILC-3^c^		6.02	0.32	2.60	

a Calculated by the elemental analysis results, and l-proline content was calculated according to the following formula: 
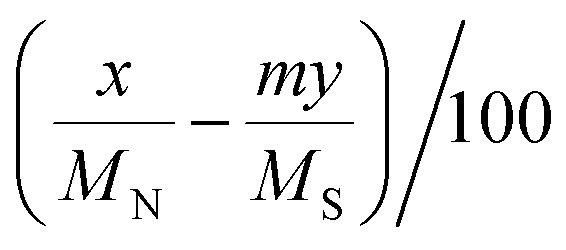
, *x* represents N content and *y* represents S content, 
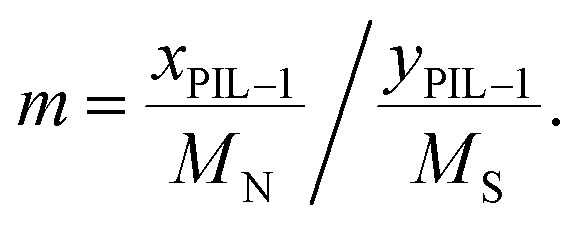

b Calculated according to the following formula: 
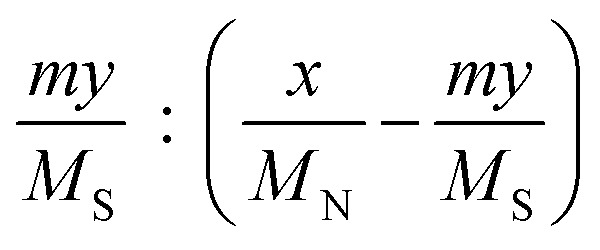
.

c PILC-3 was tested after 5 cycles determined by elemental analysis.

**Fig. 3:**
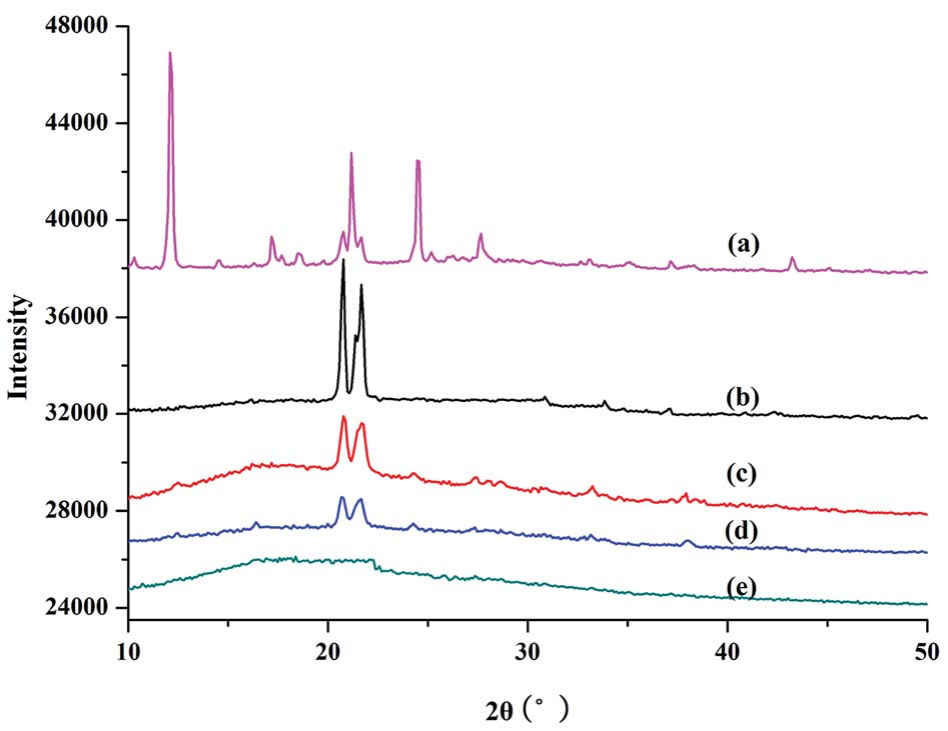
XRD patterns of (a) l-proline, (b) PILC-1, (c) PILC-2, (d) PILC-3, and (e) PILC-4.

XRD was used to study the crystal and structural changes during polymerization. Fig. 3 shows the XRD results for PILC and l-proline monomer. The main intense diffraction peaks for proline monomer appeared at 12.09° (003) and 21.17° (006), which is in accordance with a previous report concerning these amino acids (Fig. 3a).^32,33^ In Fig. 3b–d for PILC-1 and PILC-2, the diffraction peaks of l-proline had almost completely disappeared and two new peaks appeared at 20.77 and 21.67° that corresponded to average interlayer distances of *ca.* 0.42 nm.^32^ The XRD of PILC-4 revealed the disappearance of the diffraction peak corresponding to proline and there were no other peaks in the complex (Fig. 3e), which may be due to the formation of an amorphous structure and the crystal structure was completely inhibited.

**Fig. 4:**
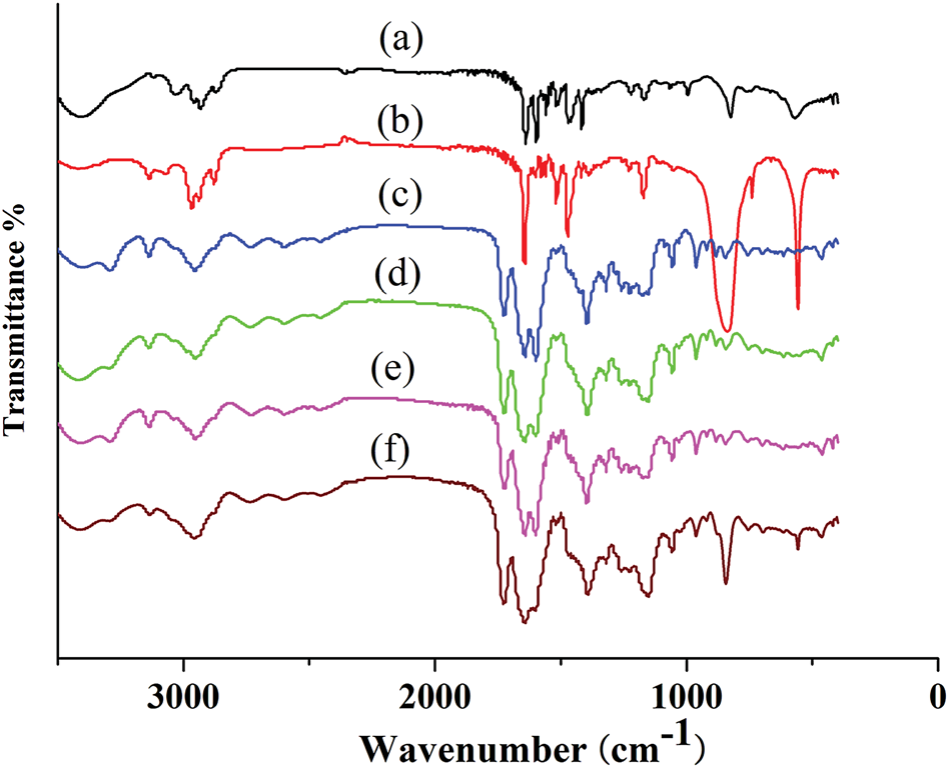
Fourier transform-infrared spectra of (a) PIL-1, (b) PIL-2, (c) PILC-1, (d) PILC-2, (e) PILC-3, and (f) PILC-4.

FT-IR spectroscopy was used to evaluate the structure of PIL complex (Fig. 4). The FT-IR spectrum of PILC (1–4) included characteristic vibration bands corresponding to C=O, –OH, and –NH at 1719, 3310, and 1050 cm^−1^ respectively (Fig. 4c–f). The characteristic band at 1625 cm^−1^ relating to the C=O of the carboxyl group of the proline unit and the band at 1600 cm^−1^ associated with the C–N of PIL-1 appeared in the spectrum of PILCs (Fig. 4a*vs.*Fig. 4c–e), suggesting formation of the complex was successful. The bands at about 2947 cm^−1^ corresponded to the asymmetric stretching vibration of the N–H which also proved that l-proline exited in the polymer complex network (Fig. 4c–f). In addition, because of the formation of hydrogen bonds or ionic interactions between the carboxyl groups of l-proline and ionic groups of PIL, the bands at 3310 cm^−1^ corresponding to the asymmetric stretching vibration of the OH group in l-proline also shifted to a lower position.^24^ Meanwhile, the characteristic band at 850 cm^−1^ was related to the P–F of the PF_6_^−^ group (Fig. 4b*vs.*Fig. 4f), which proved that PIL-2 exited in PILC-4.

**Fig. 5:**
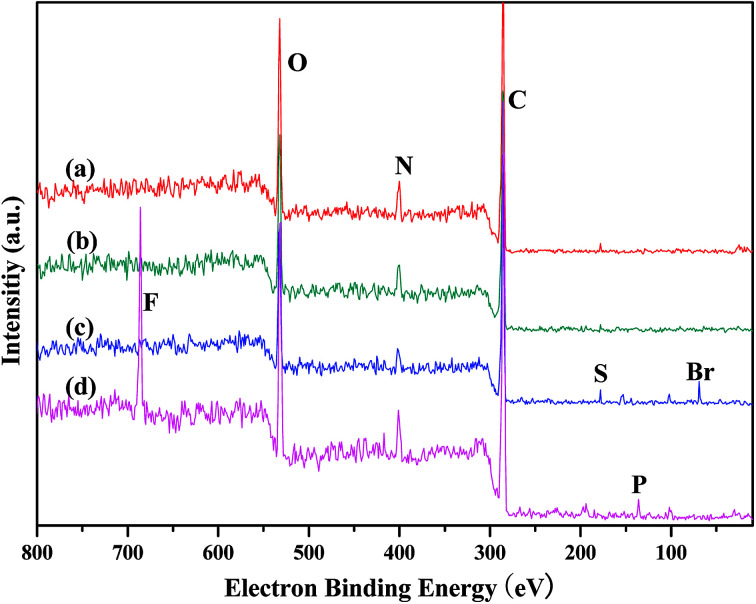
XPS of (a) PILC-1, (b) PILC-2, (c) PILC-3, and (d) PILC-4.

**Scheme 1:**
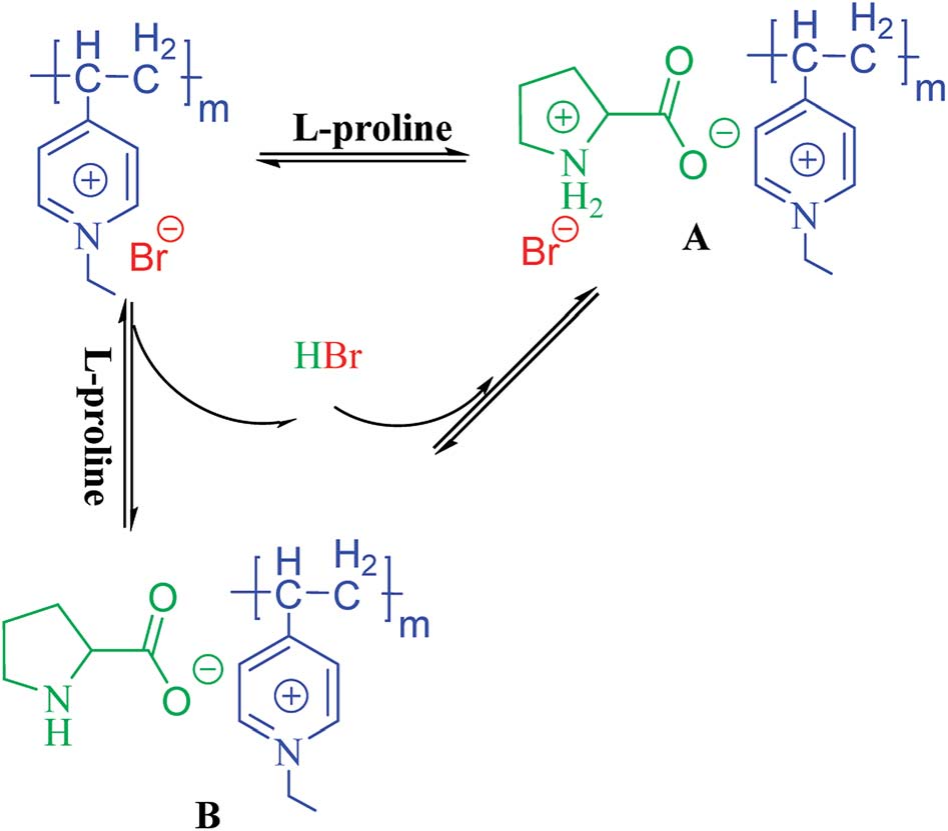
Species for the direct interaction of (l)-proline with PIL-1.

Elemental analysis (Table 1) of the PILCs revealed they had a stoichiometric composition in accordance with the experimental ratios. In addition, the load of the catalyst of the heterogeneous system can be obtained by elemental analysis. XPS spectra were used to determine the surface chemical properties of the PILCs, as shown in Fig. 5. No characteristics peaks of Br in PILC-1 and PILC-2 are found which suggests an anion exchange on PIL-1 with the carboxylate of (l)-proline (species B, Scheme 1) but not seems to an effective solvating the (l)-proline by PIL-1 (species A, Scheme 1).^34^ Br was in PILC-3, which can be attributed to the addition of a high ratio of PIL-1. F and P had also appeared in PILC-4 due to the anion exchange on PIL-2 with carboxylate of (l)-proline and more PIL-2 exiting in complex network.

### Catalytic performance

A typical aldol reaction between 4-nitrobenzaldehyde and acetone catalyzed by l-proline, proline pyridinium salt (synthesis in ESI†) and the complex network system (10 mol% of 4-nitrobenzaldehyde) in DMF was stirred at 25 °C for 24 h. The reaction results are presented in Table 2. The catalytic activity of the PILCs was significantly higher than that of l-proline and proline pyridinium salt. The conversion rates of PILC-1, PILC-2, and PILC-3 were above 80%, while the conversion rates of the small molecule catalyst (l-proline) and proline pyridinium salt were only 55% and 35%. The reaction rates demonstrate that the PIL network catalysts have the excellent catalytic activity. Compared to l-proline (76% ee value) and proline pyridinium salt (71% ee value), the 77% ee value of PILC-3 indicated that the optical activity of l-proline was maintained in the polymer network. The decreasing ee of the other PILCs (PILC-1, PILC-2, and PILC-4) revealed the flaws in the heterogeneous catalytic system.^10^ We also added an equal quantity of PIL-1 to l-proline DMF solution to study the effect of PIL on catalytic performance. However, the catalytic activity and enantioselectivity were unaltered compared with the pure l-proline. These results suggest the interactions between PIL and l-proline had no effect on catalytic performance in a free solution state. Compared to proline pyridinium salt, the improved catalytic activity and enantioselectivity of PILC-3 can be attributed to the newly confined complex network structure and the synergistic effect of the PIL. This also highlights the capacity of PIL additive to solvate l-proline molecule avoiding proline aggregates and favoring increasing the catalytic activity. By using a large excess of PIL, the supramolecular interactions result in a homogeneous dispersion for l-proline in the polymer network, to which PIL provides an appropriate mass transfer for the catalytic process.

**Table 2:** Aldol reaction between acetone and 4-nitrobenzaldehyde catalyzed by different catalyst systems for 24 h in DMF

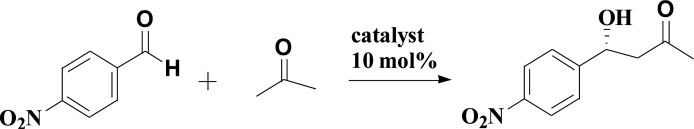			
Catalysts	Temperature (°C)	% conversion^a^	% ee^b^
l-Proline	25	55	76
Proline pyridinium salt	25	35	71
PIL-1 + l-proline (mol ratio 1 : 1)	25	55	78
PILC-1	25	80	60
PILC-2	25	80	65
PILC-3	25	82	77
PILC-4	25	70	37

a Determined by ^1^H NMR spectroscopic analysis of the product.

b Determined by HPLC using a chiral column.

Furthermore, the new system was used to catalyze the relatively complicated three-component reactions of 4-chlorobenzaldehyde with 2-hydroxy-1,4-naphthoquinone and 3-amino-5-4-methylpyrazole. Heterocyclic compounds are very important organic compounds widely used in the fields of medicine,^36^ pesticides,^37^ and other materials that are synthesized by MCRs.^38,39^ PILC-1 was first used to catalyst three-component reactions in an acetonitrile, ethanol, and THF system (Table 3). Interesting, catalytic activity and enantioselectivity were the highest for the PILC-1 in ethanol as it had the optimal conversion (79%) and ee value (91%) among the solvents (Table 3, entry 3).

**Table 3:** Effect of solvents on reactions at 80 °C for 21 h

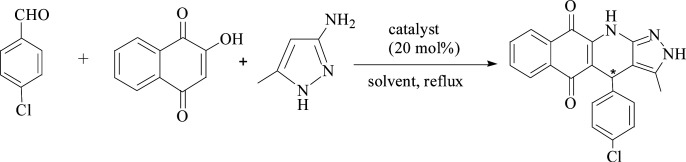				
Entry	Catalyst	Solvent	Conversion (%)	ee (%)
1	PILC-1	THF	55	64
2	PILC-1	MeCN	70	92
3	PILC-1	EtOH	79	91

**Scheme 2:**
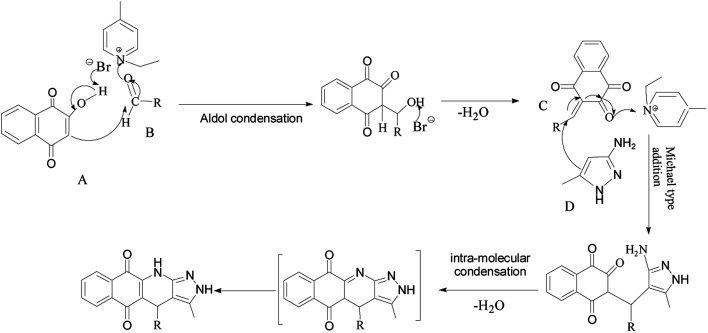
Proposed mechanism for the synthesis of 2*H*-benzo[*g*]-pyrazolo[3,4-*b*]quinoline-5,10(4*H*,11*H*)-diones.

To determine the effect of different structures on the catalytic properties of PILCs, three component reactions were carried out with different catalyst systems (Table 4). l-Proline, which had a 70% conversion and 75% ee value at 21 h, and proline pyridinium salt (69% conversion and 67% ee value) were used as the reference (Table 4, entry 1 and entry 2). The addition of PIL to the reaction had no obvious effect on the catalytic activity and enantioselectivity of l-proline as the conversion was 71% and the ee value was 75%. PILC-1 had better catalytic activity and enantioselectivity (75% conversion rate and 91% ee) than l-proline and proline pyridinium salt, which showed that the layer structure provided a more favorable catalytic environment and improved the catalytic properties. PILC-2 (72% conversion rate and 97% ee) displayed higher selectivity than PILC-1. PILC-3 had the best conversion rate (92%) and ee value (98%), meaning that more PIL-1 has the synergic effects and improved the catalytic properties. The polymer network acted not only as a better support for l-proline, but also as a promoter to promote the catalytic activity and enantioselectivity. The catalysts combining the merits of the synergic effects of PIL and the confined effects of this polymer network provided efficient heterogeneous catalytic systems. However, PILC-4 displayed poor catalytic activity (60% conversion rate), but good enantioselectivity (99% ee). The hydrophobic PIL structure did not aid in the reaction substrate entering the system and, thus, inhibited the reaction. An advantage of PILCs as catalysts is that the catalytic properties can be adjusted by changing the PIL anion. In addition, PILC-3 shows the excellent catalytic properties, a plausible mechanism for PILC-3 catalyzed one-pot MCR is outlined in Scheme 2.^40,41^ As a donor, the Br anion of the IL activated the O–H of A, and the cation of the IL activated the C=O of B as a electronic acceptor. This synergistic effect of the PIL led to the addition of one molecule of A to one molecule B. The next step involved a Michael addition of D to the C=C bond of C, which was also activated by the anion of the ionic liquid, leading to the target compound with high efficiency. In this catalytic procedure, the cation and the anion of PIL-1 have a synergetic effect on the substrates. This is a possible reason why PILC-3 could catalyze MCRs more effectively.

**Table 4:** Comparison of different catalyst systems in three component reactions between 4-chlorobenzaldehyde, 2-hydroxy-1,4-naphthoquinone, and 3-amino-5-4-methylpyrazole at 80 °C in ethanol

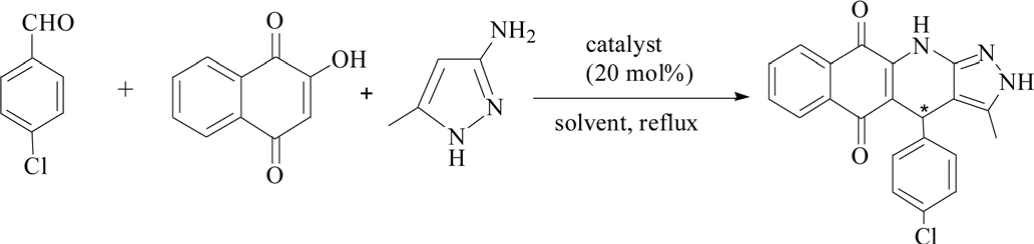				
Entry	Catalyst	Reaction time (h)	Conversion^a^ (%)	ee^b^ (%)
1	Proline	21	70	75
2	Proline pyridinium salt	21	69	67
3	Proline + PIL-1	21	71	75
4	PILC-1	21	79	91
5	PILC-2	21	72	97
6	PILC-3	21	**92**	**98**
7	PILC-4	21	60	**99**

a Determined by ^1^H NMR spectroscopic analysis of the product.

b Determined by HPLC using a chiral column.

The three-component reactions were also extended to other several substituted benzaldehydes, including 2-hydroxy-1,4-naphthoquinone and 3-amino-5-4-methylpyrazole, and the results are presented in Table 5. PILC-3 effectively promoted reactions with a variety of aromatic aldehydes (*e.g.*, *p*-nitrobenzaldehyde), where the products had better conversion (97%) and ee value (87%) in ethanol than in MeCN (Table 5).

**Table 5:** The three component reactions between different substitution at 80 °C with PILC-3 as catalyst

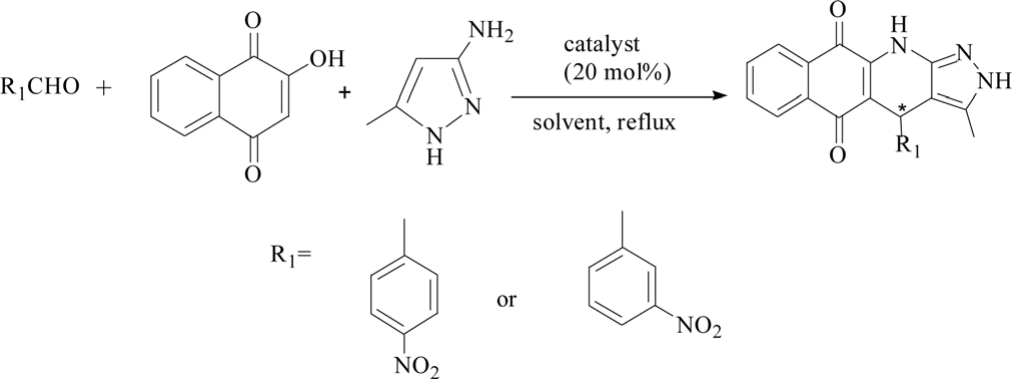				
R_1_	Solvent	Reaction time (h)	Conversion (%)	ee (%)
*p*-Nitrobenzaldehyde	EtOH	21	97	87
*m*-Nitrobenzaldehyde	EtOH	21	70	82
*p*-Nitrobenzaldehyde	MeCN	21	85	74
*m*-Nitrobenzaldehyde	MeCN	21	80	68

**Fig. 6:**
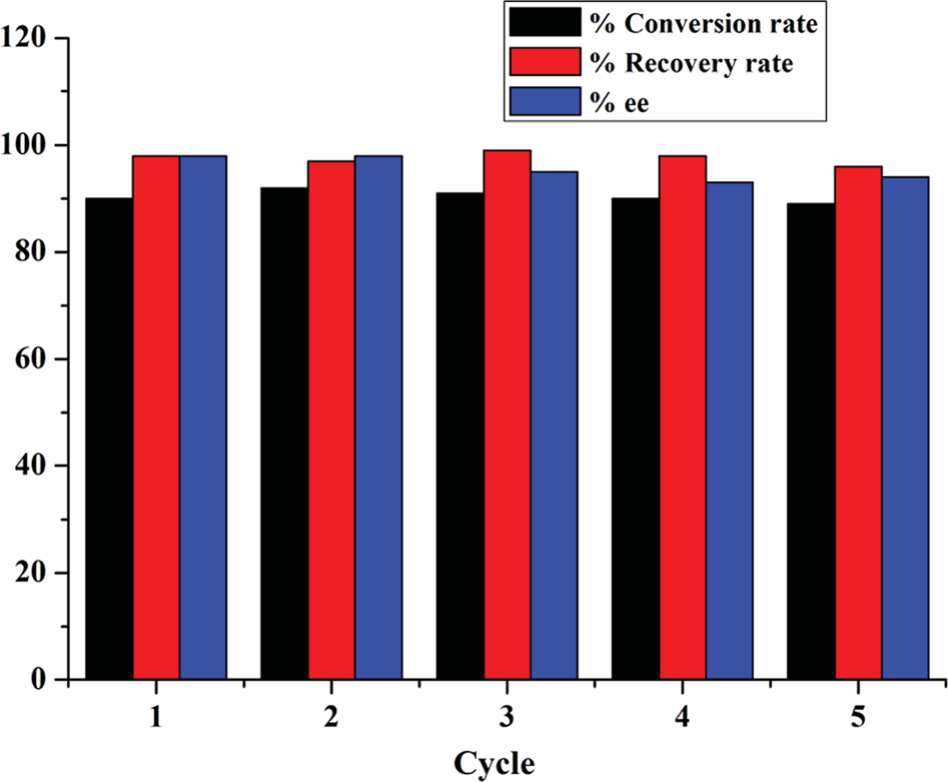
Three component reactions between 3-amino-5-4-methylpyrazole, 2-hydroxy-1,4-naphthoquinone, and 4-chlorobenzaldehyde catalyzed by PILC-3 in ethanol for 21 h in multiple cycles.

Furthermore, PILC-3 was successfully used in the three component reaction as a catalyst for 5 cycles with no discernible decrease in activity and enantioselectivity (Fig. 6). The elemental analysis result also proved that l-proline had no loss during consecutive cycles (Table 1).

## Conclusions

A new heterogeneous proline catalyst system was prepared based on the strong noncovalent bonds between PIL and l-proline and the further free radical polymerization of l-proline monomer. Due to the different reaction rates of PIL and l-proline, as well as different PIL anions, different polymer network structures were formed. The heterogeneous catalyst system could catalyze direct asymmetric aldol reactions and MCRs efficiently and had better catalytic activity and enantioselectivity than homogeneous counterparts. The catalytic system not only efficiently loaded the chiral catalyst simply using the noncovalent approach, the unique network structures and synergic effects of PIL also resulted in high catalytic performance for the heterogeneous l-proline. This catalytic system was able to be reused and recycled five times with no discernible loss in catalytic activity and enantioselectivity. This method of synthesizing polymer network-supported catalysts provides a new approach by which to prepare high performance supported catalysts for organic reactions.

## Conflicts of interest

There are no conflicts to declare.

## Supplementary Material

RA-008-C8RA08712A-s001
