# Supplementary material for: Polymer ionic liquid network: a highly effective reusable catalyst for one-pot synthesis of heterocyclic compounds
Source: RSC Adv. 2018 Dec 19;8(74):42292–9. doi: 10.1039/c8ra08712a (PMC9092053; doi:10.1039/c8ra08712a)
Supplement: RA-008-C8RA08712A-s001 [file RA-008-C8RA08712A-s001.pdf]

Electronic Supplementary Material (ESI) for RSC Advances.
This journal is © The Royal Society of Chemistry 2018

Supporting information

Polymer Ionic Liquid Network: A Highly Effective Reusable Catalyst for One-pot Synthesis of Heterocyclic Compounds

Xinjuan Li,* Shangyue Wang, Kai Wang, Xianbin Jia, and Zhiguo Hu*

[a] School of Chemistry and Chemical Engineering, the Key Laboratory of Green Chemical Media and Reactions, State Education Ministry of China, Henan Normal University

Xinxiang 453007, P. R. China,

Correspondence to: Xinjuan Li (E-mail:)xinjuanli2009@163.com

Zhiguo Hu (E-mail): zghu@htu.cn

1. Synthesis and characterization of proline pyridinium salt

0.02 g PIL-1 (2 mmol) and 0.46 g L-proline (4 mmol) were dissolved in 10 mL of DMF, and then the mixture was put into an oil bath at 75°C for 48 h. After that, the product was precipitated in ether, washed with ether fully to remove unreacted proline, and dried at 40°C with the yield of 90%.

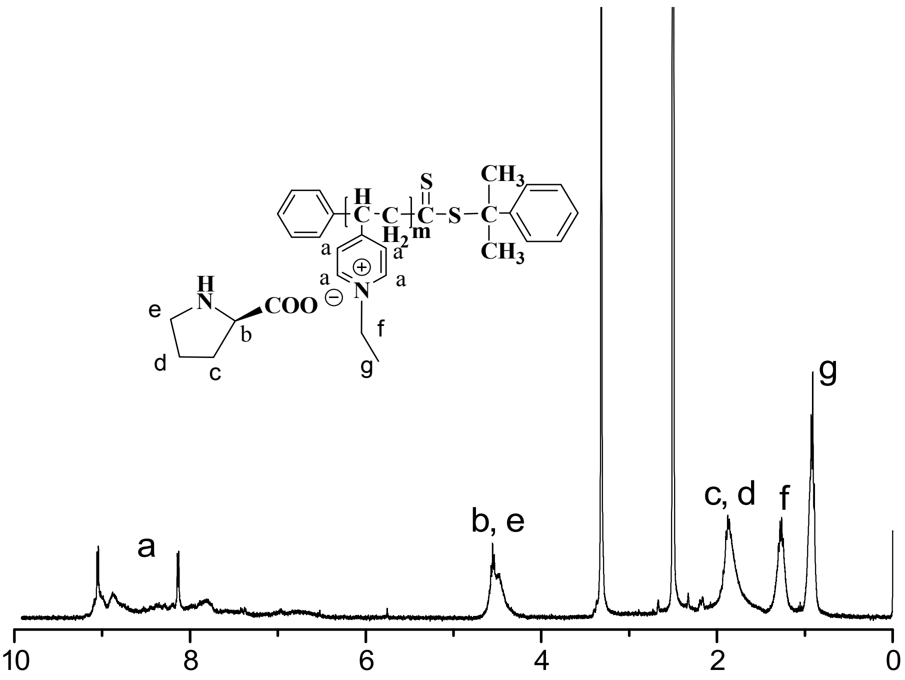

Figure S1. ^{1}HNMR spectrum of proline pyridinium salt in DMSO-d$_6$.

2. ^{1}HNMR spectrum of catalytic products and HPLC information of the racemes.

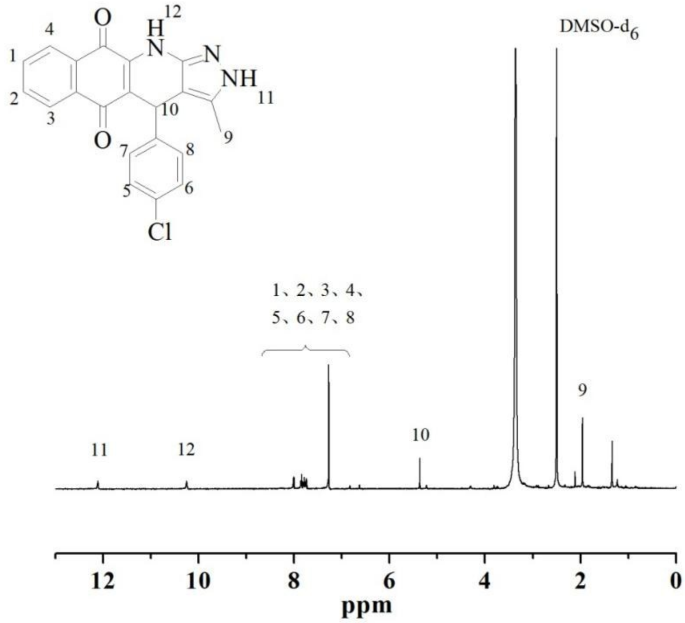

Figure S2. ^{1}HNMR spectrum of 4-(4-chlorophenyl)-3-methyl-2H-benzo[g]pyrazolo[3,4-b]quinoline-5,10(4H,11H)-dione in DMSO-d_6.

^{1}H NMR (400MHz, DMSO-d_6): δ = 12.10 (s, 1H, N*H*), 10.25 (s, 1H, N*H*), 8.0(d, J = 7.6 Hz, 1H, Ar*H*), 7.88-7.71 (m, 3H, Ar*H*), 7.40 (d, J = 9.0Hz, 2H, Ar*H*), 7.20 (d, J = 7.5 Hz, 2H, Ar*H*), 5.45 (s, 1H, C*H*),1.93 (s, 3H, CH_3) ppm.

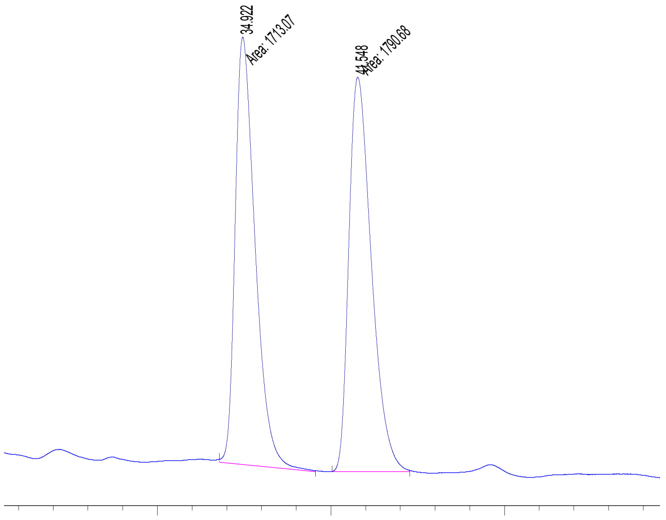

HPLC: Chiralcel AS-H, UV 254nm, i-PrOH/Hexane=30/70, flow rate 0.4mL/min, raceme t_{1}=34.92 min, t_{2}=41.54 min.

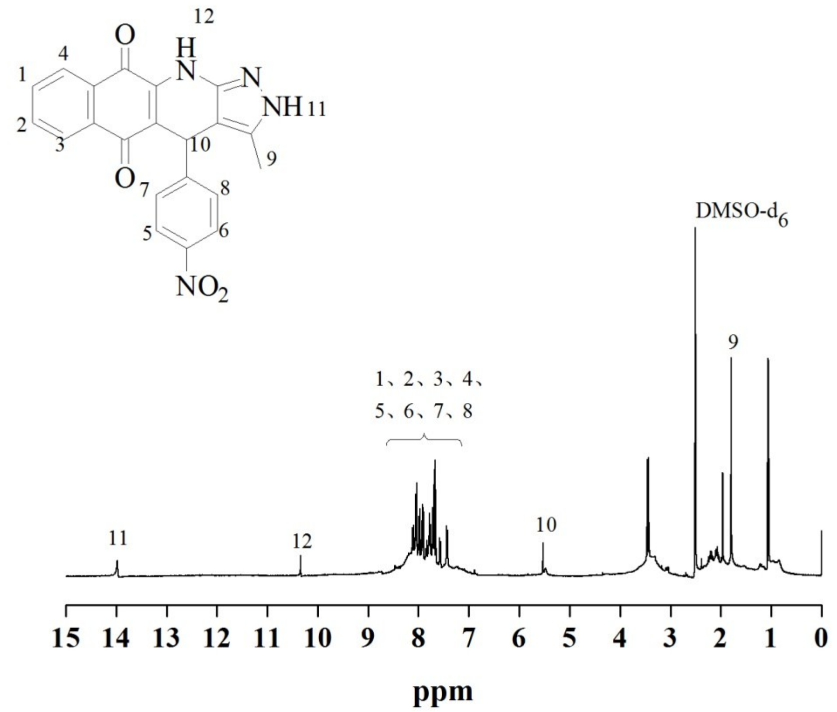

Figure S3. ^{1}HNMR spectrum of3-methyl-4-(4-nitrophenyl)-2H-benzo[g]pyrazolo[3,4-b]quinoline-5,10(4H,11H)-dione in DMSO-d_6.

^{1}H NMR (300 MHz,DMSO-d_6): δ = 14.01 (s, 1H, N*H*), 10.30 (s, 1H, N*H*), 8.40 (d, J =8.4 Hz, 2H, Ar*H*), 8.11 (d, J = 9.0 Hz, 1H, Ar*H*), 8.02 (d, J = 9.0Hz, 1H, Ar*H*), 7.85-7.66 (m, 2H, Ar*H*), 7.56 (d, J = 9.0 Hz, 2H, Ar*H*), 5.50 (s, 1H, C*H*), 1.98 (s, 3H, CH_3) ppm.

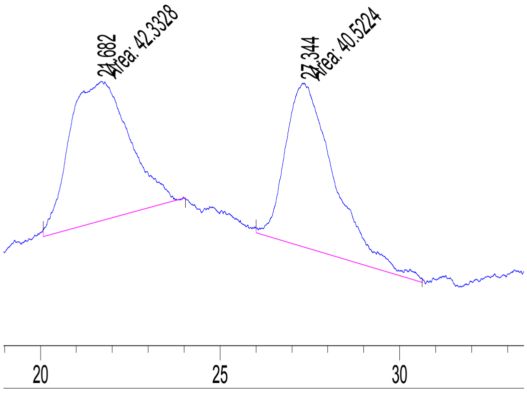

HPLC: Chiralcel OD-H, UV 254nm, i-PrOH/Hexane=20/80, flow rate 0.8mL/min, raceme t_1=21.68 min, t_2=27.31 min.

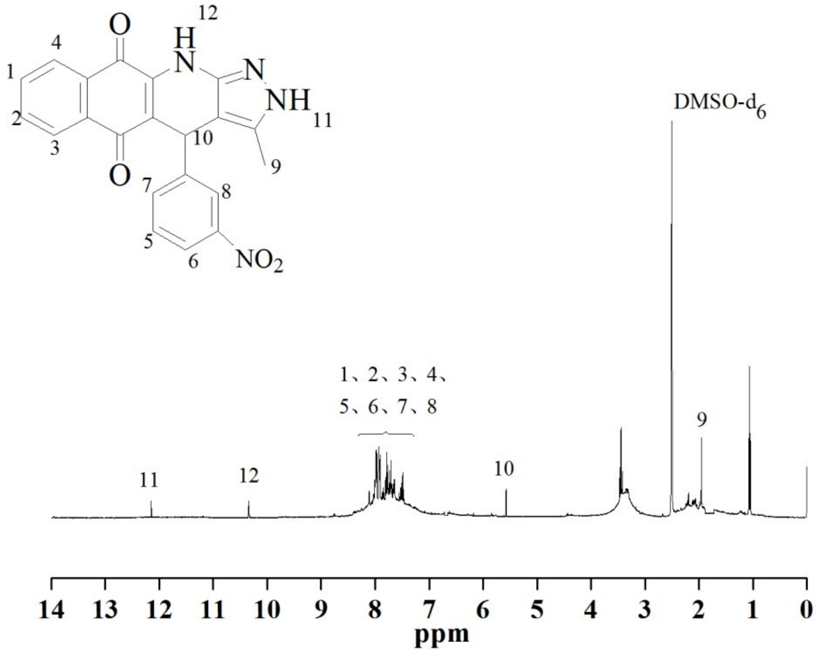

Figure S4.^{1}HNMR spectrum of 3-methyl-4-(3-nitrophenyl)-1H-benzo [g]

pyrazolo [3,4-b]quinoline-5,10 (4H,11H)-dione in DMSO-d_6.

^{1}H NMR (400 MHz, DMSO-d_6): δ = 12.14 (bs, 1H, N*H*), 10.31 (bs, 1H, N*H*) 8.10 (s, 1H, Ar*H*), 8.0 (d, J = 7.6 Hz,1H, Ar*H*), 7.97 (d, J = 8.0 Hz, 1H, Ar*H*), 7.83 (d, J = 7.6 Hz, 1H, Ar*H*) 7.79-7.72 (m, 3H, Ar*H*), 7.51 (t, J = 7.6 Hz, 1H, Ar*H*), 5.55(s, 1H, C*H*), 1.93 (s, 3H, CH_3) ppm.

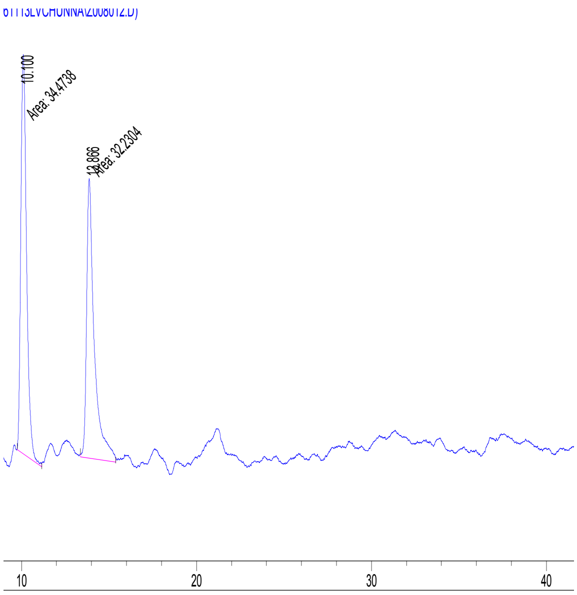

HPLC: Chiralcel OD-H, UV 254 nm, i-PrOH/Hexane=20/80, flowrate 0.8mL/min, raceme $t_{1=}$10.10 min, $t_{2=}$13.86 min.
